# Genome-wide identification, phylogeny and expression analysis of the *SPL* gene family and its important role in salt stress in *Medicago sativa* L.

**DOI:** 10.1186/s12870-022-03678-7

**Published:** 2022-06-15

**Authors:** Fei He, Ruicai Long, Chunxue Wei, Yunxiu Zhang, Mingna Li, Junmei Kang, Qingchuan Yang, Zhen Wang, Lin Chen

**Affiliations:** Institute of Animal Science, Chinese Academy of Agricultural Sciences, Beijing, 100193 China

**Keywords:** *Medicago sativa*, *SPL* gene family, Salt stress, Legume

## Abstract

**Background:**

SQUAMOSA promoter-binding protein-like (SPL) transcription factors are widely present in plants and are involved in signal transduction, the stress response and development. The *SPL* gene family has been characterized in several model species, such as *A. thaliana* and *G. max*. However, there is no in-depth analysis of the *SPL* gene family in forage, especially alfalfa (*Medicago sativa* L.), one of the most important forage crops worldwide.

**Result:**

In total, 76 putative *MsSPL* genes were identified in the alfalfa genome with an uneven distribution. Based on their identity and gene structure, these *MsSPLs* were divided into eight phylogenetic groups. Seventy-three *MsSPL* gene pairs arose from segmental duplication events, and the *MsSPLs* on the four subgenomes of individual chromosomes displayed high collinearity with the corresponding *M. truncatula* genome. The prediction of the *cis*-elements in the promoter regions of the *MsSPLs* detected two copies of ABA (abscisic acid)-responsive elements (ABREs) on average, implying their potential involvement in alfalfa adaptation to adverse environments. The transcriptome sequencing of *MsSPLs* in roots and leaves revealed that 54 *MsSPLs* were expressed in both tissues. Upon salt treatment, three *MsSPLs* (*MsSPL17*, *MsSPL23* and *MsSPL36*) were significantly regulated, and the transcription level of *MsSPL36* in leaves was repressed to 46.6% of the control level.

**Conclusion:**

In this study, based on sequence homology, we identified 76 *SPL* genes in the alfalfa. The *SPLs* with high identity shared similar gene structures and motifs. In total, 71.1% (54 of 76) of the *MsSPLs* were expressed in both roots and leaves, and the majority (74.1%) preferred underground tissues to aerial tissues. *MsSPL36* in leaves was significantly repressed under salt stress. These findings provide comprehensive information regarding the SPB-box gene family for improve alfalfa tolerance to high salinity.

**Supplementary Information:**

The online version contains supplementary material available at 10.1186/s12870-022-03678-7.

## Background

Alfalfa (*Medicago sativa* L.) is the most widely grown forage legume crop worldwide [1]. Alfalfa has been widely used in animal feed because of its high biomass yield, good palatability, and strong adaptability. Currently, almost half of the world’s irrigated land and approximately 20.0% of cultivated land are affected by salinity [2]. However, many plant growth areas, such as China, are on saline-alkali soil, which seriously affects the growth and development of alfalfa. Transcription factors (TFs) play extremely important roles in controlling the growth and development of plants. TFs greatly affect plant development, secondary metabolism, and abiotic stress tolerance by binding *cis*-acting elements in the promoter regions of target genes [3, 4]. Although the *SPL* gene can regulate inflorescence formation and fruit development and enhance stress resistance, knowledge regarding the *MsSPL* gene in alfalfa is limited. Therefore, it is important to explore the possible functions of *MsSPL* genes to understand the regulation of growth, development, and abiotic stress in alfalfa.

*SPLs* constitute a plant-specific family and are widely distributed in green plants. *SPL* is a general term for a type of transcription factor, and its structure is similar to the SBP box [5]. The SQUAMOSA promoter-binding protein (SBP) domain is highly conserved, with a length of approximately 76 amino acids [6]. The SBP domain contains two tandem zinc fingers (Cys-Cys-His-Cys and Cys-Cys-Cys-His) and one nuclear localization signal (NLS) motif [6, 7]. The members of this transcription factor family share a highly conserved DNA binding domain, the SBP. The SBP box was first isolated from the *A. majus* cDNA library, and because of its ability to recognize and bind the SQUAMOSA (SQUA) promoter, it was named SQUA [8]. An increasing amount of evidence suggests that TFs play an important role in the regulatory network of plant growth and development [9]. Various gene families, such as TEOSINTE BRANCHED 1, CYCLOIDEA, PCF1 (*TCP*) [10], and IQ67-Domain (*IQD*) [11], have been found in eukaryotes.

With the publication of many plant genomes, *SPL* gene family members has been identified and characterized in *A. thaliana* [12], *O. sativa* [13], and *G. max* [14]. According to the sequence homology and hylogenetic analyses of *SPL* genes, this family is usually divided into 6–9 subgroups. In *P. trichocarpa*, there are 28 *PtSPL* genes, and these genes are divided into 8 subgroups [15]. In *A. thaliana*, a total of 16 members have been identified as SPL proteins and named *AtSPL1* to *AtSPL16*. They were divided into eight groups according to the amino acid sequence [16]. The functions of these *SPL* genes in *A. thaliana* have also been identified, and these genes play an important role in leaf, flower, and shoot development [17, 18].

MicroRNAs are small RNAs of approximately 16–26 nucleotides in length that regulate gene expression at the posttranscriptional level in a sequence-specific manner [19]. As a key regulatory factor in most biological processes, the miR156/SPL module participates in the transformation from the vegetative stage to the reproductive stage, fruit ripening, and yield improvement [20]. However, the miR156/SPL module also responds to abiotic stresses in many plant species [21, 22]. When *A. thaliana* is under heat stress, the *SPL* gene is posttranscriptionally downregulated by miR156, which is essential for adapting to repeated heat stress [23]. The overexpression of the *SPL* gene in *B. platyphylla* is also very obvious and can improve the scavenging of reactive oxygen species to enhance tolerance to salt and drought stress [24]. Some related studies have also been carried out in alfalfa. In alfalfa, the transcript abundance of the miR156-targeted *SPL8* and *SPL13* genes was related to salt and drought tolerance [25, 26]. Studies have shown that drought stress increases the expression of miR156 by increasing leaf gas exchange and abscisic acid (ABA) while reducing water loss, thereby increasing the resistance of alfalfa to this stress [25]. To date, *SPL* genes have been isolated and identified in many plants, such as *A. thaliana* and *O. sativa*, but few studies investigated *SPL* genes in alfalfa, and the function of these proteins is unclear.

Despite the support of physiological, biochemical, and molecular data, the biological function of *SPL* transcription factor genes is still unclear. In this study, the gene structure, motif composition, chromosome location, and gene duplication of 76 recently completed alfalfa genome sequences were analyzed, and the evolutionary relationship of *M. sativa* was compared with those of *A. thaliana*, *M. truncatula*, and *G. max*. A quantitative real-time PCR (qRT–PCR) analysis was performed to examine the gene expression patterns in different tissues and their responses to salt stress. Through an overall expression analysis in alfalfa, the role of the members of the specific *SPL* gene family in the different biological processes of alfalfa was determined. This study not only provided valuable information for screening *SPL* genes important for the growth and development of alfalfa but also provided a method for mining *SPL* gene families in other plants.

## Results

### Sequence identification of the *SPL* genes in *M. sativa*

As a plant-specific transcription factor, SQUAMOSA promoter-binding protein-like (SPL) genes are involved in the plant response to adverse environmental conditions. To obtain *SPL* genes in the forage legume alfalfa, the SBP domain, a canonical feature of SPL, was screened from a Chinese landrace (*Medicago sativa* L. XinJiangDaYe) genome using HMM and BLASTP [27]. The hits were then confirmed by Pfam (http://pfam.xfam.org/) and the Conserved Domain Database (CDD) (https://www.ncbi.nlm.nih.gov/cdd). Ultimately, 76 genes were designated *MsSPL* and named *MsSPL1—MsSPL76* (Table S1)*.*

The prediction of the subcellular location showed that Ms*SPLs* were localized in the nucleus and that 16 (21.1%) SPLs were also distributed in the cytoplasm, suggesting that the putative *MsSPL* transcription factor*s* function mainly in the nucleus. The predicted MsSPLs vary in terms of the protein length and isoelectric point (pI), ranging from 100 (*MsSPL*42) to 1,026 (*MsSPL*76) amino acids (aa) and 5.24 (*MsSPL42*) to 9.65 (*MsSPL49*), respectively (Table S1).

### Sequence alignment and phylogenetic analysis of *MsSPLs*

The multiple alignment showed that in addition to the conserved SBP domain (approximately 78 amino acid residues in length), most MsSPLs shared a highly conserved nuclear localization signal (NLS) and two zinc finger-like structures, namely, Zn-1 and Zn-2 (Fig. S1). In Zn-1, the CCCH is at positions 214, 219, 236, and 239, while in Zn-2, the CCHC is at positions 255, 258, 262, and 274 amino acids. Consistent with a previous report [28], the predicted NLS possesses conserved residues, such as lysine (K) and arginine (R), at positions 271–287.

**Fig. 1:**
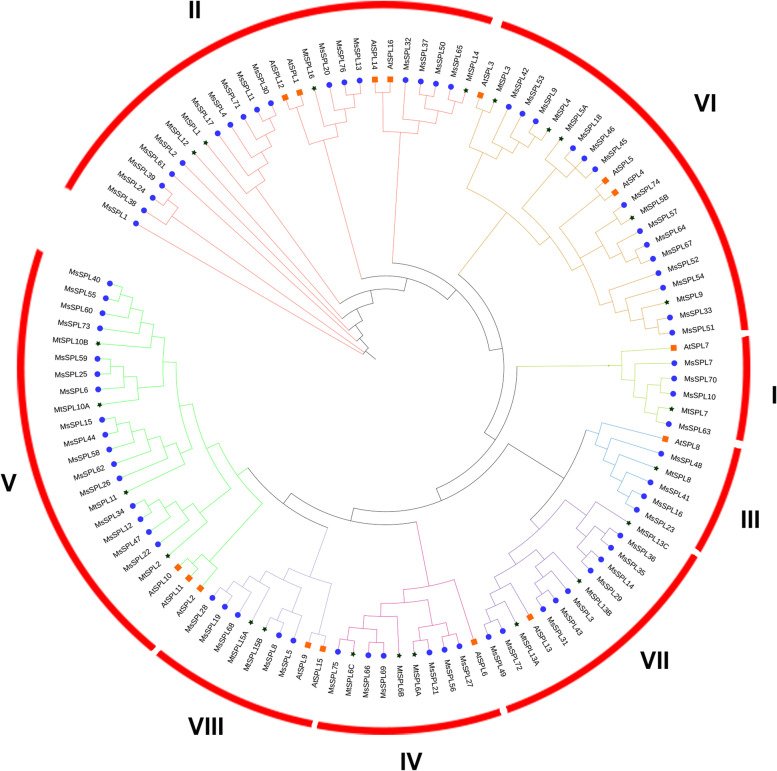
Phylogenetic analysis of SPLs from *M. sativa, M. truncatula* and *A. thaliana*. Proteins with SBP domains from *M. sativa* (*Ms*), *A. thaliana* (*At*) and *M. truncatula* (*Mt*) were searched and designated as SPL. Neighbor-joining (NJ) was used for the unrooted phylogenetic tree with the maximum likelihood method (1000 bootstraps)

To analyze the phylogenetic relationship, a neighbor-joining tree of SPLs in *M. sativa* (76), *M. truncatula* (23) and *A. thaliana* (16) was constructed using MEGA (Fig. 1). The MsSPLs similar to their counterparts in *M. truncatula* and *A. thaliana* were clustered into eight groups (Groups I ~ VIII) with varying numbers of members. The largest group (Group II) contained 18 members, accounting for 23.7% of the MsSPLs, whereas the smallest groups (Group I and Group III) possessed four members. Relative to *A. thaliana* SPLs, the homologs in the two legumes (*M. sativa* and *M. truncatula*) are closer. Interestingly, compared with MtSPL, multiple (2–6) MsSPL counterparts were grouped into the same cluster, indicating the expansion of MsSPL, probably due to genome duplication of the tetraploid forage.

### Gene structure and motif composition of *MsSPLs*

**Fig. 2:**
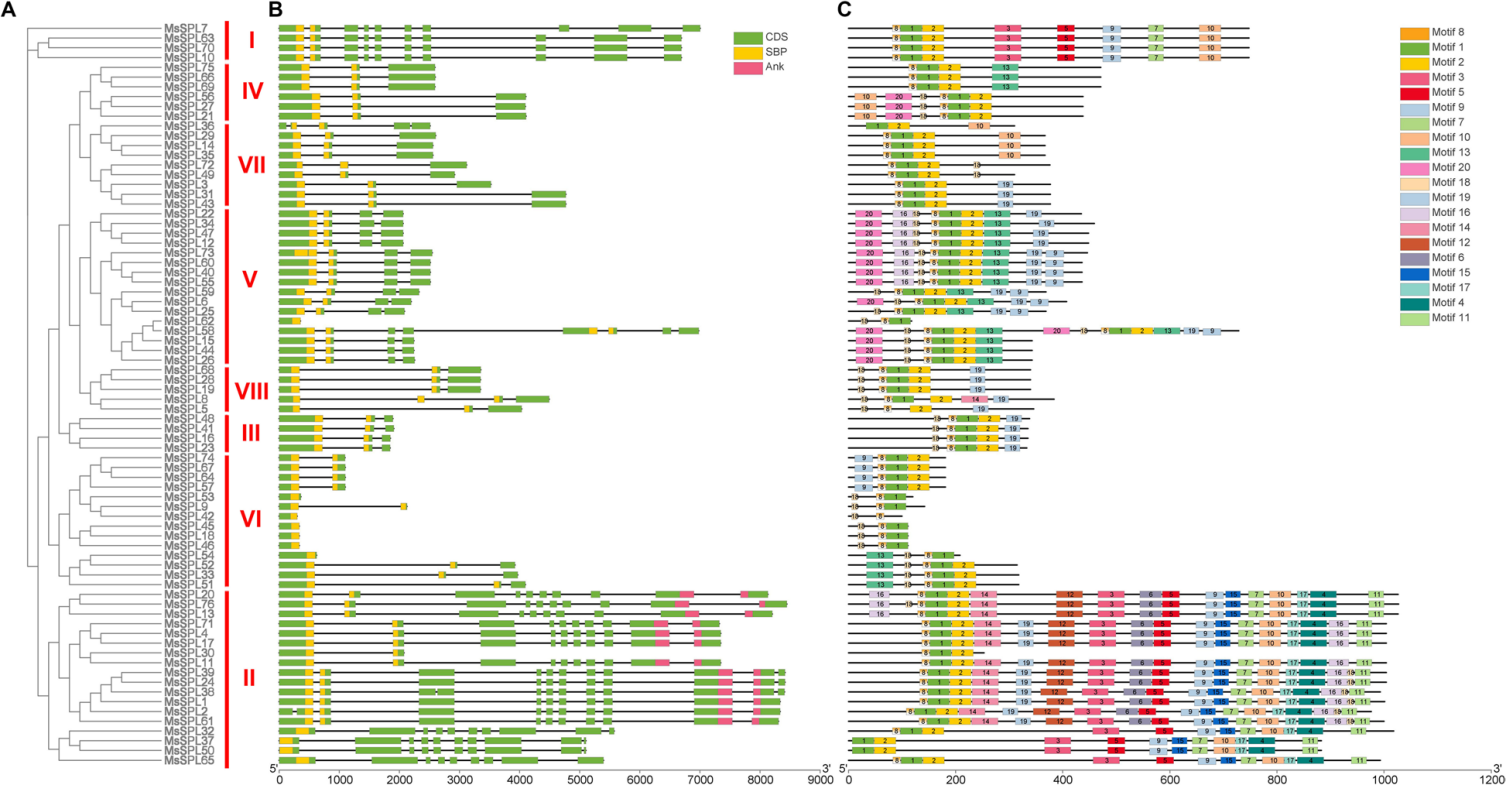
Analysis of the gene structure and conserved motifs of *MsSPL* based on phylogenetic relationships*.* **A** A phylogenetic tree was constructed based on the full-length sequences of *M. sativa* SPL proteins. **B** Exon/intron structure of *MsSPLs*. The black lines indicate introns. **C** Motif composition of putative MsSPL proteins. Motifs are displayed by colored boxes numbered 1–20. CDS stands for coding

A phylogenetic tree was constructed based on the predicted full-length MsSPL protein sequences and these proteins were also roughly divided into eight subgroups (Fig. 2A). An analysis of the gene structure of the *SPL* family in alfalfa revealed that the number of exons varied from 1–11. It seems that the *MsSPL* members in one group share a similar number of exons, with difference in intron sizes (Fig. 2B). Approximately half (52.6%) of the *MsSPLs* consist of 3–4 exons, of which 34.2% (26 out of 76) with 3 exons and 18.4% (14 out of 76) with 4 exons (Fig. 2B). The members of Groups I and II, excluding *MsSPL30,* contain relatively more exons (10–12) than the average number of exons in *MsSPLs*. The *MsSPLs* in Group VI comprise no more than three (1–3) exons. In addition to the conserved SBP domain at the N-terminus of MsSPLs, there is a conserved ankyrin (ANK) domain at the C-terminus of some Group II members (70.6% = 12/17), which is involved in protein–protein interactions in *G. max* [14]. The findings were consistent with a previous report in *S. miltiorrhiza* [29]. Therefore, *MsSPLs* from the same group share a similar gene structure, and the length of the exons is conserved correspondingly.

We examined the motif composition of the MsSPLs using MEME. In total, 20 motifs (motifs 1–20) were identified in the 76 putative MsSPLs ranging from 2 (MsSPL42) to 18 (MsSPL1, MsSPL2, MsSPL24, MsSPL38, MsSPL39 and MsSPL61) for individual proteins (Fig. 2C). On average, Groups II and VI contained the most and the fewest motifs, respectively. Among them, motifs 1, 2 and 8 were the top three motifs present in 97.4%, 89.5% and 96.1% of the MsSPLs, respectively, suggesting that these motifs are the most important components of MsSPL proteins. Similar to the gene structure, the MsSPL members from the same group, particularly Group I, shared similar motif compositions, including the motif type and number. Some motifs were present in certain groups. For example, motifs 3, 5 and 7 were present exclusively in the SPL members in Groups I and II (Fig. 2C). Among the Group II members, 13 contain a conserved motif, and 6 contain 18 of the 20 motifs, except for Motif 13 and Motif 20. Therefore, MsSPL members of the same group share a similar gene structure and motif composition, while SPLs from different groups are likely to have specific structures, implying that the functional conservation and diversity of the MsSPLs evolved during evolution.

### Analysis of the distribution, gene duplication and synteny of the *MsSPLs*

**Fig. 3:**
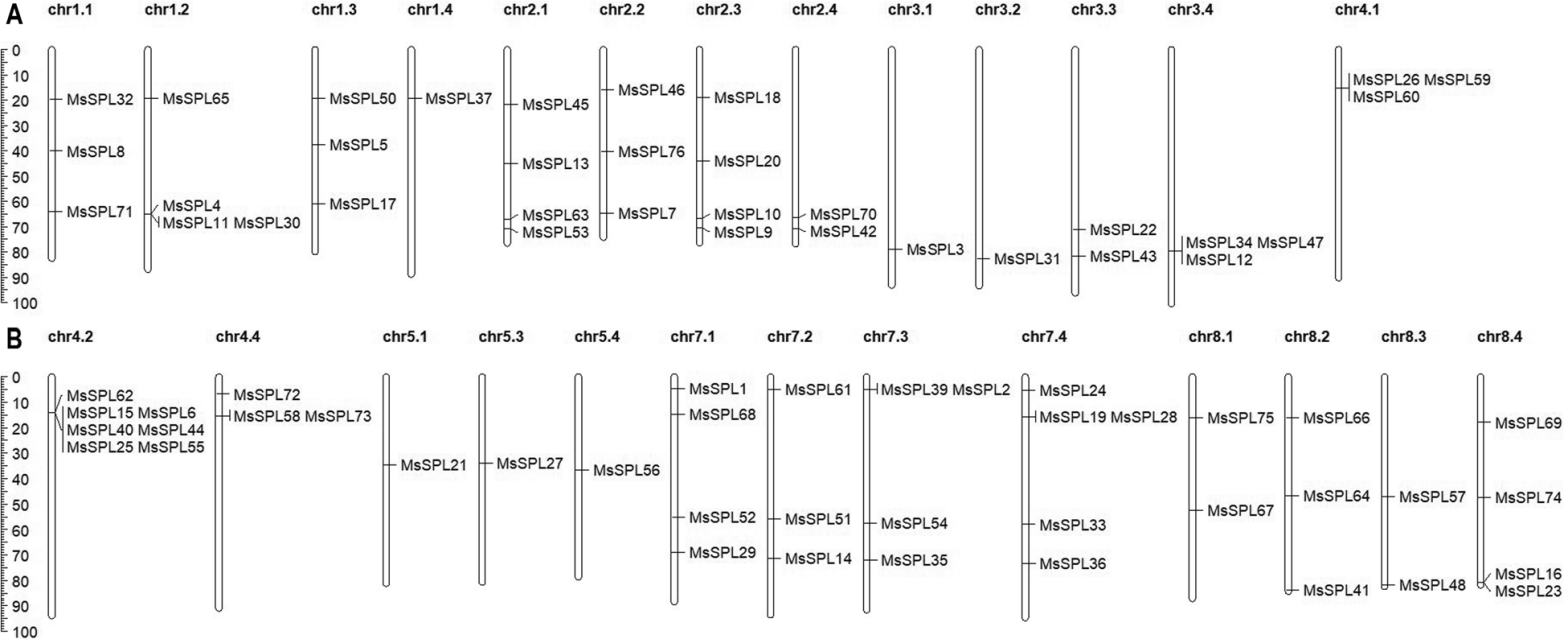
Distribution of *SPL* genes in the *M. sativa* genome. **A** Location of genes on chr1.1 to 4.1. **B** Location of genes on chr4.2 to 8.4. Chromosomes are indicated in different colors and numbered on the top. The scale (Mb) represents the lengths of the chromosomes

The predicted *MsSPLs* were mapped based on the genome database of *M. sativa* (XinJiangDaYe) [27]. Seventy-four of the 76 *MsSPLs* were unevenly distributed on 26 chromosomes, and the remaining two (*MsSPL*38 and *MsSPL*49) have not yet been assembled (Fig. 3). On average, there are approximately 2.8 *SPL* genes on each chromosome (Chr). Among them, no *SPL* was identified on Chr4.3, Chr5.2 or Chr6, while Chr4.2 possessed seven *SPLs*, probably due to gene duplication events.

**Fig. 4:**
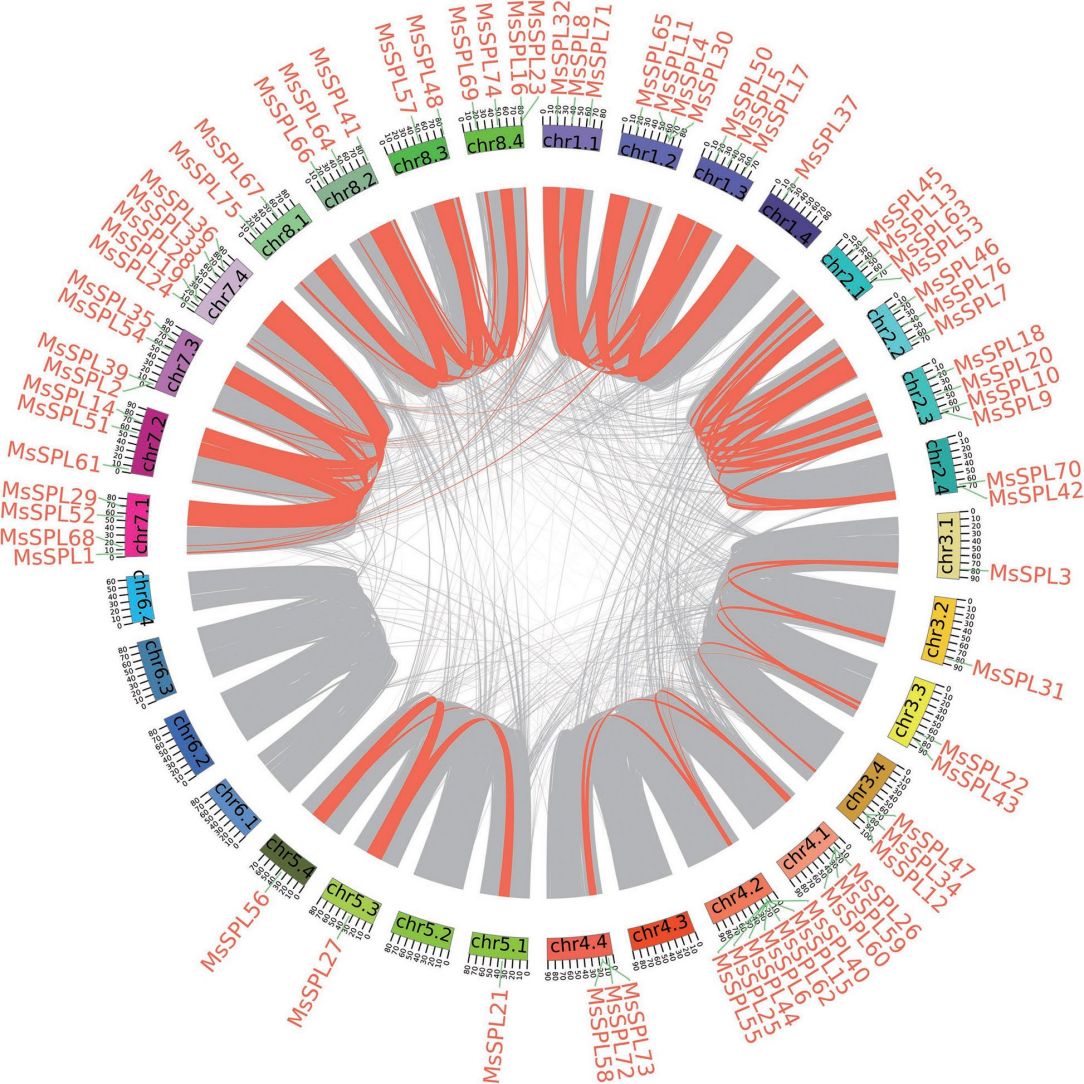
Schematic representation of the interchromosomal relationship among the *MsSPLs*. Syntenic blocks in the alfalfa genome are indicated by lines in orange

The analysis of the duplication event in the *MsSPL* family showed that there were 73 pairs of segmental duplicates but no tandem duplications (Fig. 4). *MsSPL* homologs (such as *MsSPL*8 and *MsSPL*68) from different chromosomes share higher identity. This result suggests that segment duplication may contribute to *MsSPL* expansion.

### Evolutionary analysis of the *MsSPLs* and orthologs from three model species

**Fig. 5:**
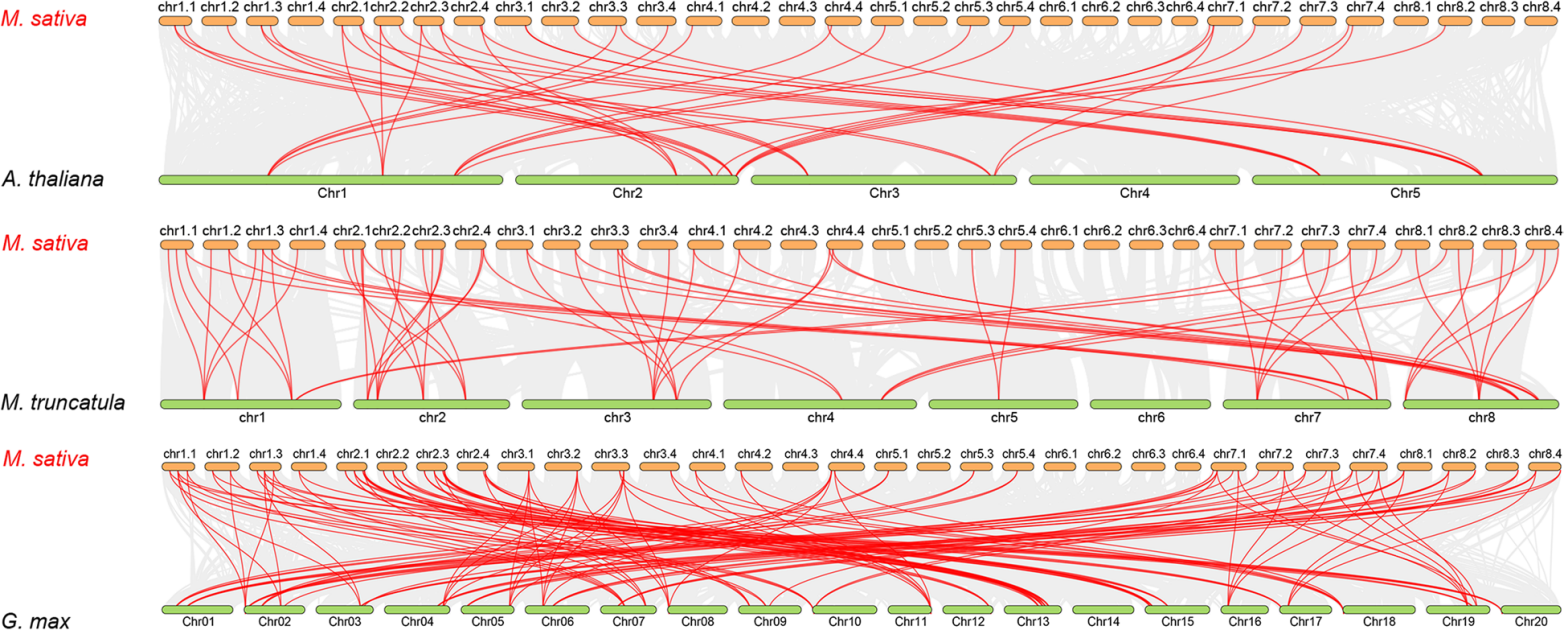
Synteny analysis of *SPL* genes between *M. sativa* and three representative plant species. Gray lines in the background indicate collinear blocks within *M. sativa* and the indicated plant, whereas the red lines highlight syntenic *SPL* gene pairs

To explore the evolutionary origin of *MsSPLs*, we performed a syntenic analysis of *SPLs* from three model species *A. thaliana*, *M. truncatula* and *G. max*. In total, 16, 23, and 41 *SPLs* have been identified in *A. thaliana* (125 Mb) [30], *M. truncatula* (500 Mb) [31] and *G. max* (1.025 Gb) [32]. In total, 57 *MsSPL*s displayed syntenic relationships with *M. truncatula*, 56 *MsSPL*s displayed syntenic relationships with *G. max* and 33 *MsSPL*s displayed syntenic relationships with *A. thaliana* (Fig. 5). Among these *MsSPLs*, 131 pairs of orthologous genes were found with *G. max*, 79 pairs of orthologous genes were found with *M. truncatula*, and 40 pairs of orthologous genes were found with *A. thaliana*. Consistent with *the MsSPL* distribution on the chromosome, chromosomes 1, 2 and 7 of alfalfa accounted for the top three *SPL* homologous gene pairs with the three model plants (Table S2). Syntenic blocks between the two Medicago species showed that the four subgenomes of alfalfa had high collinearity with the corresponding *M. truncatula* genome. Approximately 63.3% (50 of 79) of the *MsSPLs* paired with *MtSPLs* on the same chromosome, suggesting the relatively conserved distribution of *SPLs* between the two legumes. The pairing of 36.7% of the *SPLs* across chromosomes implies the occurrence of interchromosomal rearrangements, particularly between chromosome 4 and chromosome 8, as reported by Li et al. [33].

### Prediction of the *cis*-acting elements in the promoter of* MsSPLs*

**Fig. 6:**
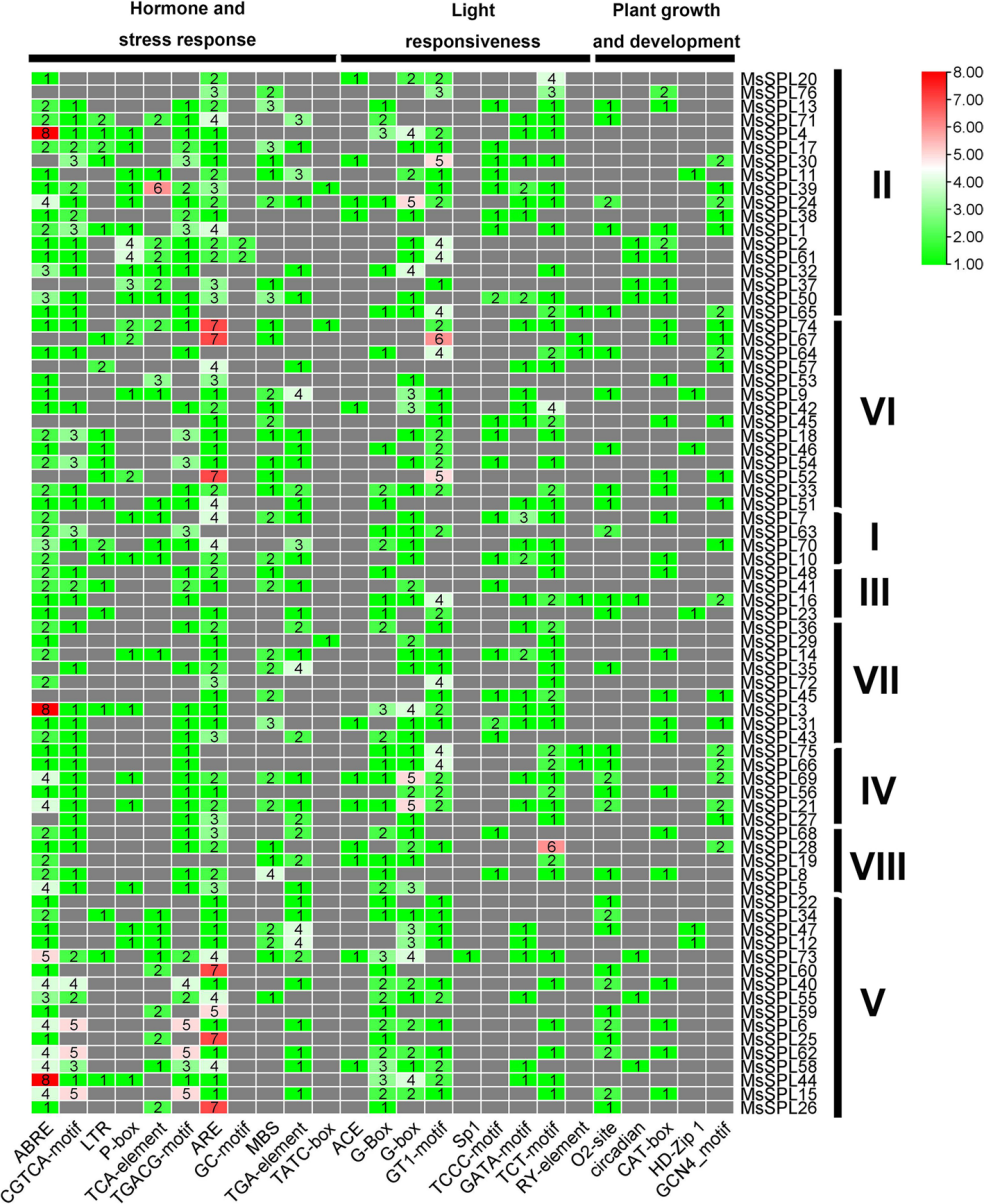
Analysis of *cis*-acting elements in the putative promoter of *MsSPLs*. The number in color represents the copy of the *cis*-acting element (at the bottom) of individual *MsSPLs* (on the right)

The *cis*-elements 2 kb upstream of the start codon (ATG) of *MsSPLs* were analyzed using the PlantCARE database (http://bioinformatics.psb.ugent.be/webtools/plantcare/html/). According to the classification of wheat [34], 25 *cis*-elements were found for *MsSPLs,* with 11 (44.0%) related to hormone and stress responses, nine (36.0%) related to light responsiveness, and five (20.0%) related to plant growth and development (Fig. 6 and Table S3). Regarding the *cis*-acting elements predicted to be associated with hormone and stress responses, abscisic acid (ABA)-responsive elements (ABREs) and AU-rich elements (AREs) were predominant in *MsSPLs*, accounting for 89.4% and 86.8%, respectively. Both *cis*-elements are present in individual *MsSPLs* with an average of two copies. The presence of ABRE, the major *cis*-element in ABA-responsive genes, implies the potential involvement of *MsSPLs* in alfalfa resistance to osmotic stresses, including drought and salinity, as previously reported in *O. sativa* and *A. thaliana* [23, 35].

### Comparison of the expression patterns of *MsSPLs* between roots and leaves

**Fig. 7:**
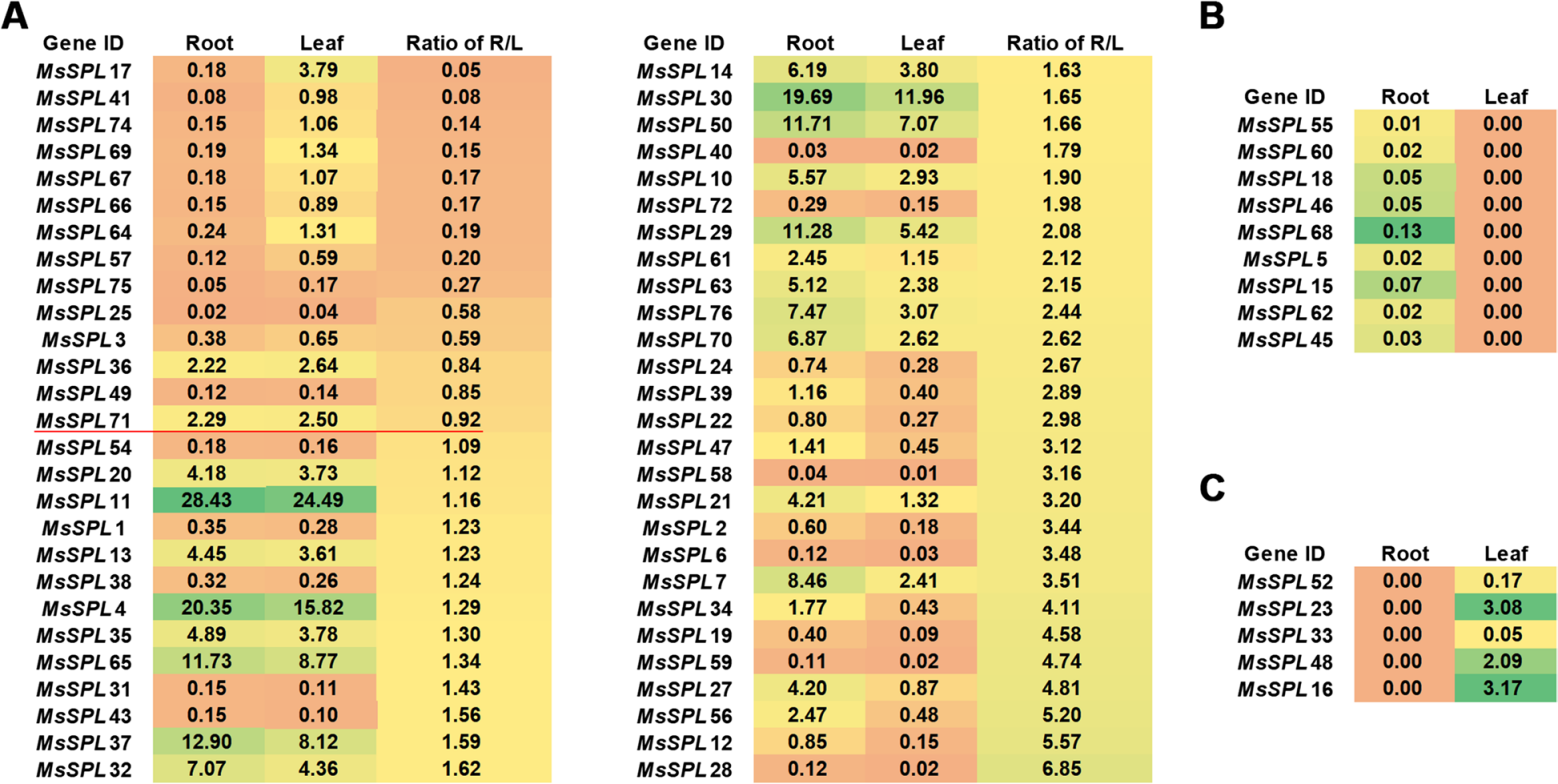
FPKM of *MsSPLs* in roots and leaves using RNA sequencing. **A** FPKM of *MsSPLs* expressed in both roots and leaves. The R/L ratio represents the ratio of FPKM in roots to that in leaves. **B** FPKM of the *MsSPLs* detected in roots but not leaves. **C** FPKM of the *MsSPLs* detected in leaves rather than roots. The color represents the FPKM normalized value. The blue and orange colors represent higher and lower expression, respectively

To compare the expression pattern of the *MsSPLs* between roots and leaves, 14-day-old alfalfa seedlings were used for RNA sequencing. Based on fragments per kilobase of transcript per million fragments mapped (FPKM), 68 *MsSPLs* were expressed in either tissue tested. Among them, 54 *MsSPLs* were detected simultaneously in both tissues, with 74.1% (40 of 54) showing higher expression in roots than in leaves (Fig. 7A). Nine MsSPLs were detected in roots rather than leaves, with four (*SPL15*, *SPL*55, *SPL*60 and *SPL*62) from Group V and two (*SPL*5 and *SPL*68) from Group VIII (Fig. 7B). In contrast, five *MsSPLs* exhibited the opposite expression pattern, showing expression in leaves but not roots (Fig. 7C). Our results show that approximately 72.1% (49 of 68) of *MsSPLs* had higher expression in roots relative to aerial tissues, indicating that these *MsSPLs* are preferentially expressed in underground tissues at this stage.

**Fig. 8:**
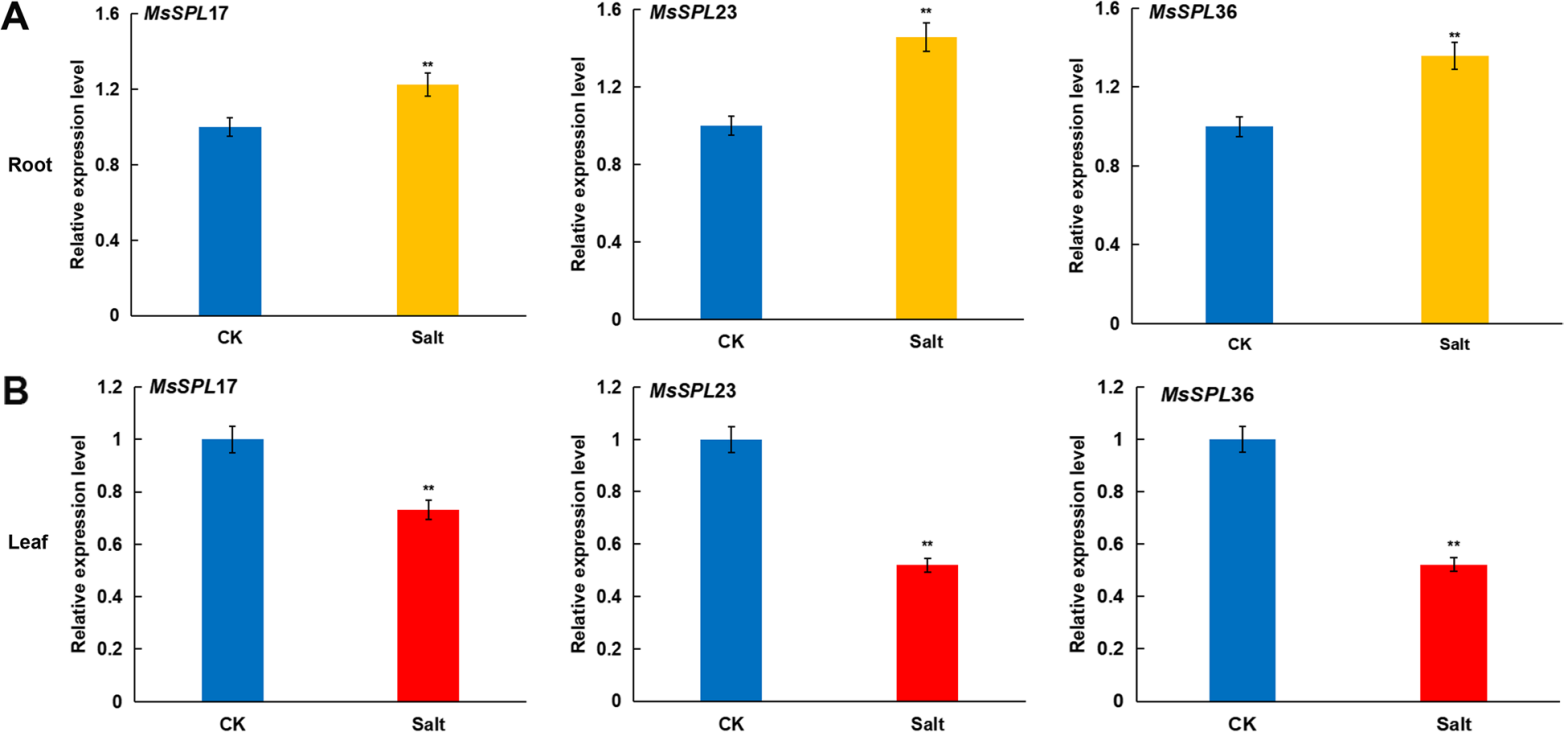
Expression levels analyses of three *MsSPL* genes under salt treatment by qRT-PCR. **A** Expression levels of three *MsSPL* genes in root. **B** Expression levels of three *MsSPL* genes in leaf. “CK” represents normal growth condition, “Salt” represents salt treatment. The levels in root and leaf of the CK were arbitrarily set to 1. Error bars represent the standard deviations of three technical replicates

To investigate the response of alfalfa to salt stress at the transcription level, we measured the expression of *MsSPLs* in roots and leaves from seedlings treated with NaCl (300 mM) for 2 weeks. The data were arbitrarily filtered by the absolute value of Log_2_(FoldChange) ≥ 1 and Padj < 0.05 (Fig. S2). Three *MsSPLs* (*MsSPL17*, *MsSPL23* and *MsSPL36*) were differentially expressed under the salt treatment, with the former *MsSPL* upregulated in the roots by 17.3% and the latter two downregulated in the leaves by 48.0% and 46.6%, respectively. Although both *MsSPL17* and *MsSPL23* showed leaf-preference under normal conditions, upon salt stress, the expression of the two genes contrasted each other, suggesting that these genes likely play opposite roles in the alfalfa response to long-term high salinity. The results were confirmed by qRT–PCR verification (Fig. 8 and Table S4).

## Discussion

*SQUAMOSA-promoter Binding* Protein Like (*SPL*s) encode a family of plant-specific transcription factors containing a conserved SQUAMOSA-promoter Binding Protein (SBP) domain and are involved in the regulation of the flowering time [36], plant development [37] and the stress response [38]. The *SPL* family has been mostly identified in model plants, such as *A. thaliana* [39], *O. sativa* [13] and *M. truncatula* [40]. The release of the alfalfa genome sequence in recent years [27, 41] has facilitated the identification of *MsSPLs* from the most valuable forage worldwide. The findings of this study could benefit alfalfa production and breeding, especially the generation of varieties with improved tolerance to environmental stresses, such as alkalinity and salinity.

*M. sativa SPLs* (*MsSPLs*) are canonical and highly conserved with *SPLs* from the model plant species. One line of evidence shows that similar to the orthologs from *M. truncatula* and *A. thaliana*, the 76 *MsSPLs* we identified here were phylogenetically clustered into eight groups based on their sequence identity, gene structure and motif composition. In individual groups, the number of MsSPLs was greater than that of *MtSPLs* or *AtSPLs,* with *MsSPL* members much closer to *M. truncatula* than *A. thaliana*. Moreover, the *MsSPLs* from the four subgenomes of alfalfa showed high collinearity with *M. truncatula* orthologs from the corresponding chromosome, and neither species had an *SPL* gene on chromosome 6 [40]. Multiple *MsSPLs* are probably attributed to the genome duplication of the tetraploid legume in comparison with *MtSPLs*. Interestingly, although there were 73 pairs of segment duplications among the *MsSPLs*, no tandemly duplicated *MsSPL* pairs were found (Fig. 4), indicating that the two legumes diverged prior to the occurrence of genome recombination for *SPLs*. Notably, in addition to the conserved motifs, such as Motif 1, Motif 2 or Motif 8, several unique motifs were present in certain groups of *MsSPLs*, suggesting a potential contribution to the specific biological functions of the dedicated *MsSPL* groups.

It appears that *MsSPL36* is a crucial candidate for improving alfalfa tolerance to salt stress. Plant *SPLs* are well known as targets of microRNA156 in regulating phase change (from the vegetative to reproductive stage) and the stress response [3, 42]. For example, miR156-mediated downregulation of three *SPL*s, i.e., *SPL2, SPL9* and *SPL11,* enhanced *A. thaliana* tolerance to heat stress [23]. Thirty-one maize *SPL*s displayed variations in their expression behavior upon exposure to one or more stresses, such as dehydration, salinity, cold and ABA [38], and transgenic tobacco expressing ZmmiR156 exhibited improved performance against drought and salt [42]. In addition, the *SPLs* in *T. chinensis* and *D. glomerata* have been shown to respond to salt, drought and heat stresses via the negative control of miR156 [21, 43]. In China, most alfalfa plantations are located in regions with saline-alkali soil due to the lack of farmland [44]. An urgent and promising task for alfalfa production in the nation is to breed varieties with tolerance to high salinity [45]. Our prediction of the *cis*-elements present in the putative promoter region of the *MsSPLs* revealed that 89.4% of *MsSPLs* possessed ABA-responsive elements (ABREs) [46], suggesting a possible contribution to the alfalfa abiotic stress response. Similar results have been documented in wheat and *Betula luminifera* [34, 47]. The transcriptomic analysis revealed that although 10.5% of the *MsSPLs* were undetected in our experiments, 89.5% of the detected *MsSPLs* were expressed in either the roots or leaves of 2-week-old plants, with 74.1% preferring roots to leaves. Under long-term high salinity (0.3 M NaCl) pressure, three *MsSPLs* were differentially expressed with *MsSPL17* up-regulated in roots, while *MsSPL23* and *MsSPL36* down-regulated in leaves of two-week-old plants. Our verification by quantitative RT–PCR showed that *MsSPL36* in leaves was repressed by the salt treatment to 46.6% of the control level. Experimental evidence is needed to support the notion that *MsSPL36* is involved in the alfalfa response to environmental stresses, especially salt. The generation of transgenic alfalfa with *MsSPL*36 knockout via miR156 or overexpression could facilitate the elucidation of its functions in forage under adverse environmental conditions. A comprehensive analysis with multiple time points of salt treatment could be helpful for a dynamic illustration of *MsSPL* expression profiles in response to stress.

## Conclusion

The phylogeny and diversification of *SPL* genes in alfalfa were investigated at different levels, including gene structures, evolutionary relationships, synteny analysis and expression patterns. All 76 *MsSPL* genes were divided into 8 groups, and genes in the same group shared similar evolutionary features and expression patterns, implying potentially similar functions for *MsSPL* genes. *SPLs* with a high identity shared similar gene structures and motifs. In total, 71.1% of the *MsSPLs* were expressed in both roots and leaves, and the majority (74.1%) preferred underground tissues to aerial tissues. The expression of *MsSPL36* in leaves was significantly repressed by salt stress. Our findings provide comprehensive information regarding the SPB-box gene family in alfalfa and have a certain value for alfalfa to improve salt tolerance.

## Methods

### Plant materials and growth conditions

Alfalfa seeds (Cultivar Zhongmu No. 1) from the Institute of Animal Science of the Chinese Academy of Agricultural Sciences were germinated in a petri dish, treated at 4 °C for 3 days and then grown in a greenhouse at 24 °C (day)/20 °C (night) under a 16 h light/8 h dark photoperiod at a relative humidity of 70 to 80% for 4 days. The germinated seedlings were transferred to flowerpots placed in the greenhouse and developed for 7 days. The two-week-old seedlings were irrigated either with 20 ml 300 mM NaCl solution every two days or water as a control group. After 14 days of treatment, the roots and leaves of the alfalfa seedlings were sampled, immediately placed in liquid nitrogen, and stored at -80 °C until further use.

### Identification of *SPL* genes in alfalfa

The alfalfa genome was downloaded from the alfalfa Genome Project (https://figshare.com/projects/whole_genome_sequencing_and_assembly_of_Medicago_sativa/66380). The *A. thaliana* protein sequences were obtained from *A. thaliana* Information Resource (TAIR) (https://www.arabidopsis.org/), and the *M. truncatula* genome was searched on a website (http://www.medicagogenome.org/). The largest number of *SPL* genes was screened from the alfalfa genome by two BLASTp methods, and the hidden MarKov model (HMM) profiles corresponding to the SBP domain (*PF03110*) were downloaded from the Pfam protein family database (https://pfam.xfam.org/). In total, 76 *MsSPL* genes were identified in the *M. sativa* genome using BLAST with a cutoff E-value > 1e^−9^. We collected the amino acid sequence of *A. thaliana* SPL proteins from the TAIR library, which ranges from 131 to 1035 aa. Similar *SPL* genes from the alfalfa genome were identified by using the *SPL* gene sequence of *A. thaliana* as a target. Subsequently, we analyzed the conserved domain of the *MsSPL* genes and removed the gene that did not contain the SBP conserved domain. Finally, 76 genes containing the SBP domain were screened from the alfalfa genome. The ExPASy website (https://web.expasy.org/compute_pi/) was used to analyze the *MsSPL* gene sequences to obtain the theoretical isoelectric points (pIs) and molecular weights (MWs).

### Phylogenetic analysis and intron–exon structure determination

The SPL protein sequences for the phylogenetic tree were obtained from the UniProt database (https://www.UniProt.org). The multiple amino acid sequences of identified *MsSPL* genes were aligned using Clustalx2.0 software with the default parameters. Phylogenetic trees comparing *M. sativa*, *A. thaliana* and *M. truncatula* were constructed with the NJ method, and the specific parameters were Poisson model and 1000 bootstrap replications by using the MEGA software. The SPL protein sequences from *M. sativa*, *A. thaliana* and *M. truncatula* were also aligned using the Clustalx2.0 program before the phylogenetic tree was constructed. Then, the *MsSPL* gene structure was predicted by an online gene structure editor (http://gsds.cbi.pku.edu.cn/) website to align the coding and genome sequences. The determination of the conserved motifs in the MsSPL proteins was conducted by the MEME online program (http:/meme.nbcr.net/meme/intro.html), and the parameters were set to the optimum mode width of 6 to 200 and the maximum number of motifs of 20.

### Chromosome location, gene duplication and synteny analysis

Information concerning the chromosomal location of *MsSPL* genes, including the chromosome length, gene direction, and gene start and stop positions, was obtained from the alfalfa genome database. MCScanX software was used to analyze the *MsSPL* replication events and detect collinear regions between *MsSPLs* and collinear blocks of *MsSPL* genes with *A. thaliana*, *M. truncatula*, and *G. max*. All function and chromosomal location information was obtained by TBtools software [48].

### *Cis*-element analysis

The upstream 2 kb sequence was extracted as the promoter region for the prediction of *cis*-acting elements. The homeopathy components of the promoter sequence were predicted by the online tool PlantCARE, and the predicted results were drawn by GSDS online software.

### Gene expression pattern of *MsSPL* gene families with RNA-seq data

The Illumina HiSeq 2500 platform was used to sequence the cDNA library based on synthetic sequencing technology, and a large amount of high-read data was obtained. Two replicates were prepared for the construction of a sequencing library per sample. We used RNA-seq data to analyze the gene expression patterns of *MsSPL* genes. The data were filtered and compared to the reference genome of XinJiangDaYe alfalfa. In addition, we applied FPKM (fragments per kilobase of transcript per million fragments mapped) to calculate the gene expression level according to the number of reads mapped to the reference sequence. The heatmap of the *MsSPL* gene expression profile was constructed by R software.

### Gene pattern analyses of *MsSPL* genes by real-time quantitative RT-PCR

Total RNA was extracted from the roots and leaves of normally growing and salt-treated alfalfa seedlings with TRIzol reagent according to the manufacturer's instructions. Then, the cDNA library was constructed for the subsequent reactions using the Genesand Kit (UnionScript First-strand cDNA Synthesis Mix for qPCR). SYBR Premix Ex Taq II (TaKaRa) with a CFX96 real-time PCR system (Bio-Rad) was used to conduct the RT–PCR experiments. The qRT–PCR primers were designed on the NCBI website (https://blast.ncbi.nlm.nih.gov/) (Table S4). *MsActin* was used as the internal reference gene for data normalization. A total of four samples (CK_root, CK_leaf, Salt_root and Salt_leaf) were used in this study. Three independent biological replicates and three technical repeats were taken. Roots and leaves under control conditions were selected as the control samples for measuring gene expression under salt treatment. The data were quantified by the 2^−△△CT^ method [49].

## Supplementary Information

**Additional file 1: Table S1. **List of the 76 MsSPL genes identified in this study.

**Additional file 2: Table S2. **One-to-one orthologous relationships between *Medicago sativa* L. and other plants.

**Additional file 3: Table S3. **Cis_elements contained in the MsSPL genes promoter region.

**Additional file 4: Table S4. **The primer sequences of qRT-PCR.

**Additional file 5: Fig. S1. **Alignment of the conserved SBP domain in MsSPL proteins.

**Additional file 6: Fig. S2. **Expression profile *MsSPL* genes across different tissues. 

## Data Availability

RNA sequence data from roots and leaves after 14 days of salt treatment in a greenhouse has been submitted to The NCBI Sequence Read Archive (BioProject: PRJNA777963).

## References

[1] He F, Kang JM, Zhang F, Long RC, Yang QC (2019). Genetic mapping of leaf-related traits in autotetraploid alfalfa (Medicago sativa L.). Mol Breed.

[2] Paul D, Lade H (2014). Plant-growth-promoting rhizobacteria to improve crop growth in saline soils: a review. Agron Sustain Dev.

[3] Gao J, Cui L (2014). The miR156-SPL9-DFR pathway coordinates the relationship between development and abiotic stress tolerance in plants. Plant J.

[4] Hai W, Wang H (2015). The miR156/SPL module, a regulatory hub and versatile toolbox, gears up crops for enhanced agronomic traits. Mol Plant.

[5] Liu M, Sun W, Ma Z, Huang L, Chen H (2019). Genome-wide identification of the SPL gene family in Tartary Buckwheat (Fagopyrum tataricum) and expression analysis during fruit development stages. BMC Plant Biol.

[6] Yamasaki K, Kigawa T, Inoue M, Tateno M, Yamasaki T, Yabuki T, Aoki M, Seki E, Matsuda T, Nunokawa E (2004). A novel zinc-binding motif revealed by solution structures of DNA-binding domains of Arabidopsis SBP-family transcription factors. J Mol Biol.

[7] Klein J, Saedler H, Huijser P (1996). A new family of DNA binding proteins includes putative transcriptional regulators of the Antirrhinum majus floral meristem identity gene SQUAMOSA. Mol Gen Genet.

[8] Huijser P, Klein J, Lönnig WE, Meijer H, Saedler H, Sommer H (1992). Bracteomania, an inflorescence anomaly, is caused by the loss of function of the MADS-box gene squamosa in Antirrhinum majus. Embo J.

[9] Riechmann JL, Heard J, Martin G, Reuber L, Yu GL (2001). Arabidopsis Transcription Factors: Genome-Wide Comparative Analysis Among Eukaryotes. Science.

[10] Huo Y, Xiong W, Su K, Li Y, Sun Z (2019). Genome-wide analysis of the TCP gene family in switchgrass ( Panicum virgatum L.). Int J Genomics.

[11] Yuan J, Liu T, Yu Z, Li Y, Ren H, Hou X, Li Y (2019). Genome-wide analysis of the Chinese cabbage IQD gene family and the response of Br IQD5 in drought resistance. Plant Mol Biol.

[12] Xu M, Hu T, Zhao J (2016). Developmental Functions of miR156-Regulated SQUAMOSA PROMOTER BINDING PROTEIN-LIKE (SPL) Genes in Arabidopsis thaliana. PLoS Genet.

[13] Xie K, Wu C, Xiong L (2006). Genomic organization, differential expression, and interaction of SQUAMOSA promoter-binding-like transcription factors and microRNA156 in rice. Plant Physiol.

[14] Tripathi RK, Goel R, Kumari S, Dahuja A (2017). Genomic organization, phylogenetic comparison, and expression profiles of the SPL family genes and their regulation in soybean. Dev Genes Evol.

[15] Li C, Lu S (2014). Molecular characterization of the SPL gene family in Populus trichocarpa. BMC Plant Biol.

[16] Birkenbihl RP, Jach G, Saedler H, Huijser P (2005). Functional dissection of the plant-specific SBP-domain: overlap of the DNA-binding and nuclear localization domains. J Mol Biol.

[17] Gandikota M, Birkenbihl RP, Hmann SH, Cardon GH, Saedler H (2010). The miRNA156/157 recognition element in the 3' UTR of the Arabidopsis SBP box gene SPL3 prevents early flowering by translational inhibition in seedlings. Plant J.

[18] Wu G (2006). Temporal regulation of shoot development in Arabidopsis thaliana by miR156 and its target SPL3. Development.

[19] Sun G (2012). MicroRNAs and their diverse functions in plants. Plant Mol Biol.

[20] Wu Z, Cao Y, Yang R, Qi T, Hang Y, Lin H, Zhou G, Wang ZY, Fu C (2016). Switchgrass SBP-box transcription factors PvSPL1 and 2 function redundantly to initiate side tillers and affect biomass yield of energy crop. Biotechnol Biofuels.

[21] Wang J, Ye Y, Xu M, Feng L, Xu L (2019). Roles of the SPL gene family and miR156 in the salt stress responses of tamarisk (Tamarix chinensis). BMC Plant Biol.

[22] Aung B, Gruber MY, Hannoufa A (2015). The MicroRNA156 system: a tool in plant biotechnology. Biocatal Agric Biotechnol.

[23] Stief A, Altmann S, Hoffmann K, Pant BD, Scheible WR, Bäurle I (2014). Arabidopsis miR156 regulates tolerance to recurring environmental stress through SPL transcription factors. Plant Cell.

[24] Ning K, Chen S, Huang H, Jiang J, Yuan H, Li H (2017). Molecular characterization and expression analysis of the SPL gene family with BpSPL9 transgenic lines found to confer tolerance to abiotic stress in Betula platyphylla Suk. Plant Cell.

[25] Arshad M, Feyissa BA, Amyot L, Aung B, Hannoufa A (2017). MicroRNA156 improves drought stress tolerance in alfalfa (Medicago sativa) by silencing SPL13. Plant Sci.

[26] Gou J, Debnath S, Sun L, Flanagan A, Tang Y, Jiang Q, Wen J, Wang ZY (2018). From model to crop: functional characterization of SPL 8 in M. truncatula led to genetic improvement of biomass yield and abiotic stress tolerance in alfalfa. Plant Biotechnol J.

[27] Chen H, Zeng Y, Yang Y, Huang L, Qiu Q (2020). Allele-aware chromosome-level genome assembly and efficient transgene-free genome editing for the autotetraploid cultivated alfalfa. Nat Commun.

[28] Li S, Li L, Jiang Y, Wu J, Sun H, Zhao M, Jiang Y, Zhu L, Wang Y, Su Y (2020). SQUAMOSA Promoter Binding Protein-Like (SPL) Gene Family: TRANSCRIPTOME-Wide Identification, Phylogenetic Relationship, Expression Patterns and Network Interaction Analysis in Panax ginseng CA Meyer. Plants.

[29] Zhang L, Wu B, Zhao D, Li C, Shao F, Lu S (2014). Genome-wide analysis and molecular dissection of the SPL gene family in Salvia miltiorrhiza. J Integr Plant Biol.

[30] Schneeberger K, Ossowski S, Ott F, Klein JD, Wang X, Lanz C, Smith LM, Cao J, Fitz J, Warthmann N (2011). Reference-guided assembly of four diverse Arabidopsis thaliana genomes. Proc Natl Acad Sci Belarus-Agrar Ser.

[31] Young ND, Udvardi M (2009). Translating Medicago truncatula genomics to crop legumes. Curr Opin Plant Biol.

[32] Shen Y, Liu J, Geng H, Zhang J, Liu Y, Zhang H, Xing S, Du J, Ma S, Tian Z (2018). De novo assembly of a Chinese soybean genome. Sci China-Life Sci.

[33] Li A, Liu A, Du X, Chen JY, Yin M, Hu HY, Shrestha N, Wu SD, Wang HQ, Dou QW (2020). A chromosome-scale genome assembly of a diploid alfalfa, the progenitor of autotetraploid alfalfa. Hortic Res.

[34] Zhu T, Liu Y, Ma L, Wang X, Zhang D, Han Y, Ding Q, Ma L (2020). Genome-wide identification, phylogeny and expression analysis of the SPL gene family in wheat. BMC Plant Biol.

[35] Mukherjee K, Choudhury AR, Gupta B, Gupta S, Sengupta DN (2006). An ABRE-binding factor, OSBZ8, is highly expressed in salt tolerant cultivars than in salt sensitive cultivars of indica rice. BMC Plant Biol.

[36] Wang JW, Czech B, Weigel D (2009). miR156-regulated SPL transcription factors define an endogenous flowering pathway in Arabidopsis thaliana. Cell.

[37] Cardon GH, Höhmann S, Nettesheim K, Saedler H, Huijser P (1997). Functional analysis of the Arabidopsis thaliana SBP-box gene SPL3: a novel gene involved in the floral transition. Plant J.

[38] Mao HD, Yu LJ, Li ZJ, Yan Y, Han R, Liu H, Ma M (2016). Genome-wide analysis of the SPL family transcription factors and their responses to abiotic stresses in maize. Plant Gene.

[39] Yang Z, Wang X, Gu S, Hu Z, Xu H, Xu C (2008). Comparative study of SBP-box gene family in Arabidopsis and rice. Gene.

[40] Wang H, Lu Z, Xu Y, Kong L, Han L (2019). Genome-wide characterization of SPL family in Medicago truncatula reveals the novel roles of miR156/SPL module in spiky pod development. BMC Genomics.

[41] Shen C, Du HL, Chen Z, Lu HW, Zhu FG, Chen H, Meng XZ, Liu QQ, Liu P, Zheng LH, Li XX, Dong JL, Liang CZ, Wang T (2020). The chromosome-level genome sequence of the autotetraploid alfalfa and resequencing of core germplasms provide genomic resources for alfalfa research. Mol Plant.

[42] Kang T, Yu CY, Liu Y, Song WM, Bao Y, Guo XT, Li B, Zhang HX (2020). Subtly manipulated expression of ZmmiR156 in tobacco improves drought and salt tolerance without changing the architecture of transgenic plants. Front Plant Sci.

[43] Feng G, Han J, Yang Z, Liu Q, Shuai Y, Xu X, Nie G, Huang L, Liu W, Zhang X (2021). Genome-wide identification, phylogenetic analysis, and expression analysis of the SPL gene family in orchardgrass (Dactylis glomerata L.). Genomics..

[44] Wang Y, Wang J, Guo D, Zhang H, Che Y, Li Y, Tian B, Wang Z, Sun G, Zhang H (2021). Physiological and comparative transcriptome analysis of leaf response and physiological adaption to saline alkali stress across pH values in alfalfa (Medicago sativa). Plant Physiol Biochem.

[45] Wei TJ, Jiang CJ, Jin YY, Zhang GH, Wang MM, Liang ZW (2020). Ca2+/Na+ ratio as a critical marker for field evaluation of saline-alkaline tolerance in alfalfa (Medicago sativa L.). Agronomy.

[46] Nakashima K, Yamaguchi-Shinozaki K (2013). ABA signaling in stress-response and seed development. Plant Cell Rep.

[47] Li XY, Lin EP, Huang HH (2018). Molecular characterization of SQUAMOSA PROMOTER BINDING PROTEIN-LIKE (SPL) gene family in Betula luminifera. Front Plant Sci.

[48] Chen C, Chen H, Zhang Y, Thomas HR, Xia R (2020). TBtools: an integrative toolkit developed for interactive analyses of big biological data. Mol Plant.

[49] Kenneth JL, Thomas DS (2001). Analysis of relative gene expression data using real-time quantitative PCR and the 2-ΔΔCT method. Methods.

