# Supplementary figures and images for: Genome-wide identification, phylogeny and expression analysis of the SPL gene family and its important role in salt stress in Medicago sativa L
Source: BMC Plant Biol. 2022 Jun 15;22:295. doi: 10.1186/s12870-022-03678-7 (PMC9199161; doi:10.1186/s12870-022-03678-7)

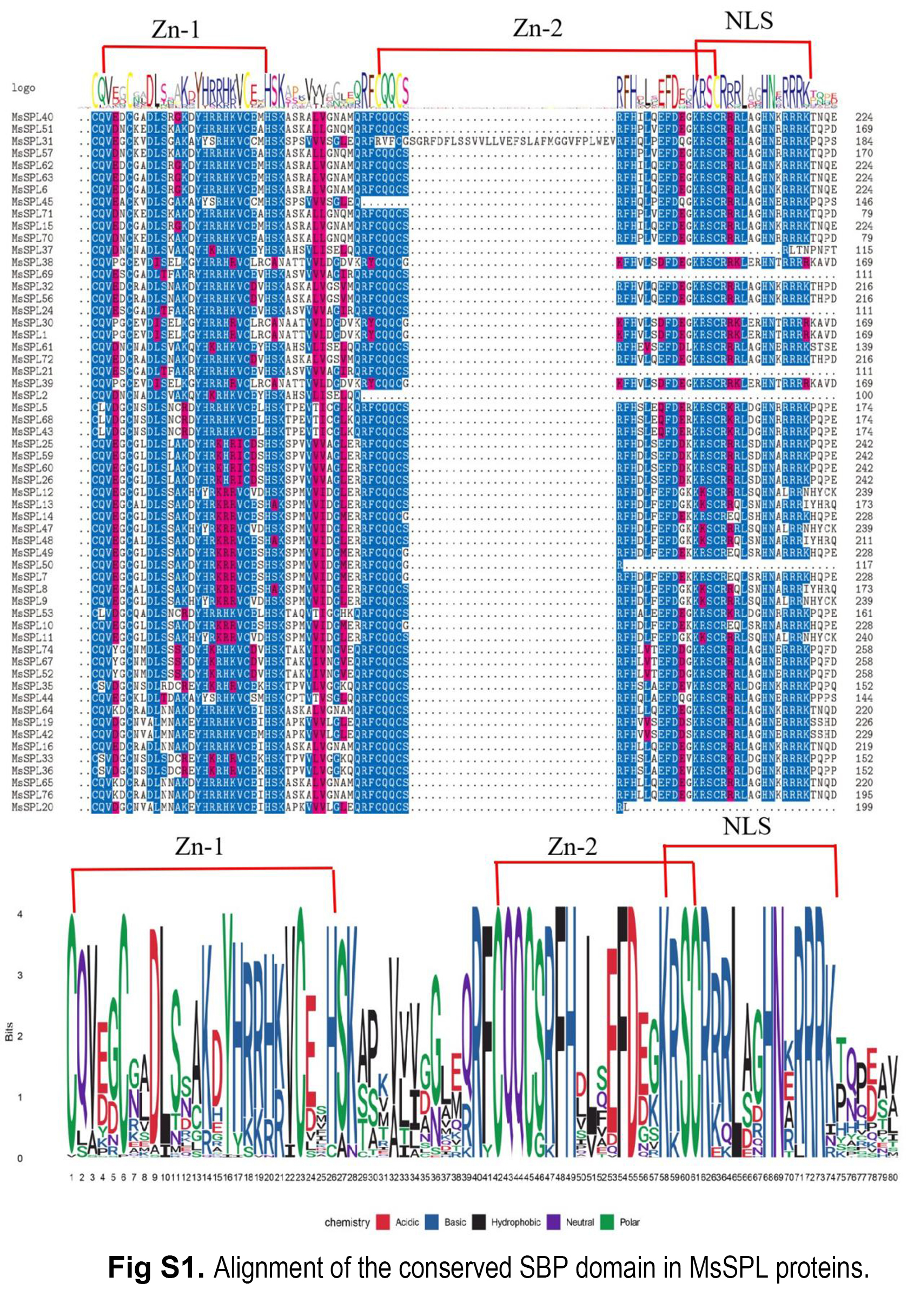

Supplement: Supplementary file 5 — Additional file 5: Fig. S1. Alignment of the conserved SBP domain in MsSPL proteins. [file 12870_2022_3678_MOESM5_ESM.jpg]

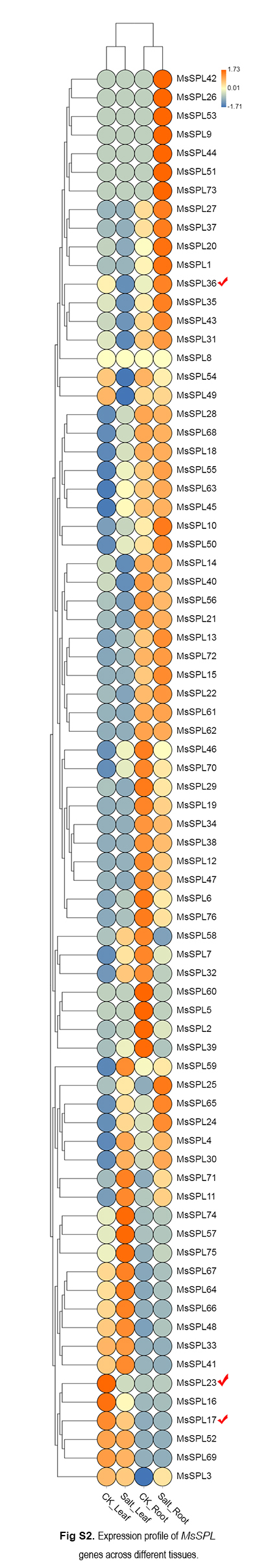

Supplement: Supplementary file 6 — Additional file 6: Fig. S2. Expression profile MsSPL genes across different tissues. [file 12870_2022_3678_MOESM6_ESM.jpg]
